# Phenotypic and Genomic Properties of *Brachybacterium vulturis* sp. nov. and *Brachybacterium avium* sp. nov.

**DOI:** 10.3389/fmicb.2018.01809

**Published:** 2018-08-07

**Authors:** Euon J. Tak, Pil S. Kim, Dong-Wook Hyun, Hyun S. Kim, June-Young Lee, Woorim Kang, Hojun Sung, Na-Ri Shin, Min-Soo Kim, Tae W. Whon, Jin-Woo Bae

**Affiliations:** ^1^Department of Life and Nanopharmaceutical Sciences, Kyung Hee University, Seoul, South Korea; ^2^Department of Biology, Kyung Hee University, Seoul, South Korea

**Keywords:** *Dermabacteraceae*, *Brachybacterium*, *Devriesea*, *Dermabacter*, *Brachybacterium vulturis* sp. nov., *Brachybacterium avium* sp. nov.

## Abstract

Two strains, VM2412^T^ and VR2415^T^, were isolated from the feces of an Andean condor (*Vultur gryphus*) living in Seoul Grand Park, Gyeonggi-do, South Korea. Cells of both strains were observed to be Gram-stain positive, non-motile, aerobic, catalase positive and oxidase negative. Growth was found to occur at 10-30°C, showing optimum growth at 30°C. The strains could tolerate up to 15% (w/v) NaCl concentration and grow at pH 6-9. The strains shared 99.3% 16S rRNA gene sequence similarity to each other but were identified as two distinct species based on 89.0-89.2% ANIb, 90.3% ANIm, 89.7% OrthoANI and 38.0% dDDH values calculated using whole genome sequences. Among species with validly published names, *Brachybacterium ginsengisoli* DCY80^T^ shared high 16S rRNA gene sequence similarities with strains VM2412^T^ (98.7%) and VR2415^T^ (98.4%) and close genetic relatedness with strains VM2412^T^ (83.3–83.5% ANIb, 87.0% ANIm, 84.3% OrthoANI and 27.8% dDDH) and VR2415^T^ (82.8–83.2% ANIb, 86.7% ANIm, 83.9% OrthoANI and 27.2% dDDH). The major fatty acid of the two strains was identified as anteiso-C_15:0_ and the polar lipids consisted of phosphatidylglycerol, diphosphatidylglycerol, presumptively phosphatidylethanolamine and three unidentified glycolipids. Strain VR2415^T^ also produced an unidentified phospholipid. The cell walls of the two strains contained *meso*-diaminopimelic acid as diagnostic diamino acid and the whole cell sugars were ribose, glucose, and galactose. The strains contained MK-7 as their predominant menaquinone. The genomes of strains VM2412^T^, VR2415^T^, and *B. ginsengisoli* DCY80^T^ were sequenced in this study. The genomic G+C contents of strains VM2412^T^ and VR2415^T^ were determined to be 70.8 and 70.4 mol%, respectively. A genome-based phylogenetic tree constructed using an up-to-date bacterial core gene set (UBCG) showed that the strains formed a clade with members of the genus *Brachybacterium*, supporting their taxonomic classification into the genus *Brachybacterium*. Based on phenotypic and genotypic analyses in this study, strains VM2412^T^ and VR2415^T^ are considered to represent two novel species of the genus *Brachybacterium* and the names *Brachybacterium vulturis* sp. nov. and *Brachybacterium avium* sp. nov. are proposed for strains VM2412^T^ (=KCTC 39996^T^ = JCM 32142^T^) and VR2415^T^ (=KCTC 39997^T^ = JCM 32143^T^), respectively.

## Introduction

The genus *Brachybacterium* was first described by Collins et al. (1988). At the time of writing, the genus contains 19 species with validly published names: *B. faecium* (Collins et al., 1988), *B. nesterenkovii* (Gvozdyak et al., 1992), *B. conglomeratum*, *B. paraconglomeratum*, *B. rhamnosum* (Takeuchi et al., 1995), *B. alimentarium*, *B. tyrofermentans* (Schubert et al., 1996), *B. fresconis*, *B. sacelli* (Heyrman et al., 2002), *B. muris* (Buczolits et al., 2003), *B. phenoliresistens* (Chou et al., 2007), *B. zhongshanense* (Zhang et al., 2007), *B. saurashtrense* (Gontia et al., 2011), *B. squillarum* (Park et al., 2011), *B. ginsengisoli* (Hoang et al., 2014), *B. huguangmaarense* (Liu et al., 2014), *B. hainanense* (Liu et al., 2015), *B. aquaticum* (Kaur et al., 2016) and *B. horti* (Singh et al., 2016). The genus *Brachybacterium* is affiliated to the family *Dermabacteraceae* with the three genera *Dermabacter*, *Devriesea*, and *Helcobacillus*. Currently, the genus *Dermabacter* is composed of three species: *Dermabacter hominis* (Jones and Collins, 1988), *D. vaginalis* (Chang et al., 2016), and *D. jinjuensis* (Park et al., 2016), while the genera *Devriesea* and *Helcobacillus* consist of a single species, *Devriesea agamarum* (Martel et al., 2008) and *Helcobacillus massiliensis* (Renvoise et al., 2009), respectively.

Strains of the genus *Dermabacter* have been classified as Centers of Disease Control and Prevention (CDC) group 3 and group 5 coryneform bacteria (Funke et al., 1994) and have been isolated from diverse clinical specimens such as cerebral abscess (Bavbek et al., 1998), blood cultures (Gómez-Garcés et al., 2001), peritoneal fluid (Radtke et al., 2001), bone deposits from an osteomyelitis patient (Van Bosterhaut et al., 2002) and fatal septicemia (Lee et al., 2011). Therefore, they are considered opportunistic pathogens. Strains of the genus *Devriesea* are involved in the pathogenesis of dermatitis, cheilitis and septicemia in lizards as facultative pathogenic bacteria (Hellebuyck et al., 2009) and strains of the genus *Helcobacillus* were isolated from human cutaneous discharge samples, showing the potential for toxicity (Renvoise et al., 2009). However, members within the genus *Brachybacterium* have not been reported to be pathogenic before now. They have been isolated from environmental samples such as coastal sand, sediment, plants, soil and seawater and from foods including milk products, cheese and salt-fermented seafood. The type strain of *B. muris* was isolated from murine liver (Buczolits et al., 2003).

Isolation of strains of *Brachybacterium* species from wide-ranging sources demonstrates that they have adapted to diverse environmental conditions using characteristics obtained in the process of diverging from other genera. Genotypic analysis based on whole genome sequences can indicate which genes are involved in the divergence and can contribute to more accurate taxonomic classification. In present study, two strains were isolated and found to belong to the genus *Brachybacterium* based on 16S rRNA gene sequences. The whole genome sequences were obtained for the newly isolated strains and from *B. ginsengisoli* DCY80^T^ as a closely related species. Although whole genome sequences of some type strains of *Brachybacterium* species are available in GenBank/EMBL/DDBJ databases, the genome sequences have not been used previously for taxonomic analysis or subjected to comparative genomic studies. Therefore, we used the available genome sequences of *Brachybacterium* species along with those of *Dermabacter* and *Devriesea* species to investigate the taxonomic status of the newly isolated strains within the genus *Brachybacterium* and to identify intra- and inter-genus differences based on genotypic characteristics.

## Materials and Methods

### Bacterial Strain Isolation

Strains VM2412^T^ and VR2415^T^ were isolated from fecal sample of an Andean condor living in Seoul Grand Park, Gyeonggi-do, South Korea (37°25^′^39.7^′′^N 127°01^′^01.2^′′^E). Strains VM2412^T^ and VR2415^T^ were isolated using a marine agar (MA) plate and a Reasoner’s 2A (R2A) medium, respectively, which were inoculated with 10^-4^ diluted fecal sample and incubated at 20°C. Tryptic soy broth (TSB) and tryptic soy agar (TSA) were used for culture at 30°C.

### 16S rRNA Gene Sequence Analysis

Genome-derived 16S rRNA gene sequences of strains VM2412^T^ and VR2415^T^ were 1,513 bp. The sequences were compared with those of other type strains using EzBioCloud server (Yoon et al., 2017). The 16S rRNA gene sequences of the two strains and type strains of closely related taxa were aligned using BioEdit (Hall, 2011). Phylogenetic trees were constructed by neighbor-joining (NJ) (Saitou and Nei, 1987), maximum likelihood (ML) (Felsenstein, 1981) and maximum parsimony (MP) (Kluge and Farris, 1969) algorithms in the MEGA6 program (Tamura et al., 2013).

### Phenotypic Characterization

Based on the 16S rRNA gene sequences, the strains were observed to share high sequence similarities (>98%) with *B. ginsengisoli* DCY80^T^ and *B. faecium* DSM 4810^T^. Therefore, these type strains were obtained from the Korean Collection for Type Cultures (*B. ginsengisoli* KCTC 29226^T^) and the Japan Collection of Microorganisms (*B. faecium* JCM 11609^T^) and used for comparison of phenotypic characteristics with the isolated strains.

Growth temperature range was determined in TSB by incubating at 4, 10, 15, 20, 25, 30, 37, 45, 55, and 70°C. The effect of salt concentration on bacterial growth was determined in TSB with 0, 1, 2, 3, 4, 5, 6, 8, 10, 12, 15, 20, 22, and 25% (w/v) NaCl. Growth in TSB at pH 4, 5, 6, 7, 8, 9, 10, and 11 was investigated for identifying pH range and optimal pH for bacterial growth. Bacterial growth was evaluated by measuring the absorbance at 600 nm (OD_600_) after incubation for 24 h, 48 h, and 1 week in the different temperature, salinity and pH conditions.

The two isolates were cultured on TSA [4% (w/v) NaCl, pH 7] and incubated at 30°C for optimal growth. After 48 h, cell morphology was identified using a light microscope (ECLIPSE 50i; Nikon) and colony morphology was determined macroscopically. Gram-staining was conducted with a Gram-staining kit (bioMérieux) according to the manufacturer’s instructions. Cell motility was identified by bacterial migration from an inoculation line in semi-solid agar with 0.4% (w/v) agar (Tittsler and Sandholzer, 1936). Bacterial survival under anaerobic conditions was determined by checking bacterial growth after incubation for 1 week in an anaerobic chamber with 90% N_2_, 5% CO_2_ and 5% H_2_.

Catalase activity was identified by bubble formation after adding drops of 3% (v/v) hydrogen peroxide solution and oxidase activity was confirmed by color transition to deep purple or blue after adding drops of 1% (w/v) tetramethyl-*p*-phenylenediamine solution (bioMérieux). Bacterial enzyme activities were examined using API ZYM and API 20 NE test strips (bioMérieux). API 20 NE test strips were also used to check the ability of bacteria to assimilate carbohydrates. Bacterial ability to ferment various carbohydrates was tested with API 50 CH test strips (bioMérieux) using API 50 CHB/E media.

For analyses of fatty acids, cell wall components, polar lipids and menaquinone, culture was performed on TSA [4% (w/v) NaCl, pH 7] at 30°C for 48 h. Bacterial fatty acid methyl esters were extracted by saponification and methylation of bacterial cells according to the protocol of the Sherlock Microbial Identification Systems (MIDI, 1999). The composition of fatty acid methyl esters was determined by gas chromatography using a calibration standard (Microbial ID). For analysis of cell wall amino acids, peptidoglycan was purified by the method of Schumann (2011). The purified peptidoglycan was hydrolyzed using 4M HCl and the hydrolysate was spotted onto a cellulose thin layer chromatography (TLC) plate with alanine, lysine, glutamic acid, glycine, aspartic acid, and *meso*-diaminopimelic acid as reference amino acids. One-dimensional TLC was performed using methanol-water-6M HCl-pyridine (80:26:4:10, v/v) as solvent. The plate was air-dried and sprayed with ninhydrin reagent. Spots were visualized by heating at 100°C for 5 min. For analysis of cell wall sugars, bacterial cells were hydrolyzed according to the protocol of Cummins and Harris (1956). The whole cell hydrolysate was spotted onto a cellulose TLC plate with ribose, arabinose, glucose, galactose, rhamnose, xylose and mannose for spot comparison. The solvent used for one-dimensional TLC development was *n*-butanol-water-pyridine-toluene (10:6:6:1, v/v). After drying, the plate was sprayed with aniline phthalate reagent and heated at 120°C for 5 min for spot detection. Polar lipids were extracted from bacterial cells and dissolved in chloroform-methanol (2:1, v/v) solution (Minnikin et al., 1984). The composition of polar lipids was determined by two-dimensional TLC using chloroform-methanol-water (65:24:4, v/v) for one dimension and chloroform-acetic acid-methanol-water (80:15:12:4, v/v) for second dimension. Total lipids were detected by spraying with 10% (v/v) ethanolic molybdophosphoric acid, then heating at 180°C for 15 min. Ninhydrin and α-naphthol reagents were used for detection of aminolipids and glycolipids, respectively. Spots were visualized by heating at 110°C for 15 min. Bacterial menaquinone was extracted from bacterial cells using chloroform-methanol (2:1, v/v) solution (Collins and Jones, 1981). The composition of bacterial menaquinones was determined by high-performance liquid chromatography (HPLC) using methanol-isopropanol (2:1, v/v) for elution.

### Whole Genome Sequence Analysis

Genomic DNA was extracted using a MG Genomic DNA Purification kit (MGmed), checked by electrophoresis in 1% agarose gel and quantified with a NanoDrop spectrophotometer (Thermo Scientific) and a Qubit fluorometer (Thermo Scientific). To generate a 20 kb library, genomic DNA was sheared with g-TUBE (Covaris) and purified using AMPure PB magnetic beads (Beckman Coulter). A total library was prepared using a DNA Template Prep Kit 1.0 (Pacific Biosciences). DNA/Polymerase Binding Kit P6 (Pacific Biosciences) was used for binding DNA template libraries to DNA polymerase P6. Whole genome sequencing was performed using the PacBio RS II sequencing platform. DNA Sequencing Kit 4.0 (Pacific Biosciences) and PacBio RS II single-molecule real-time (SMRT) Cells 8 Pac (Pacific Biosciences) were used for SMRT sequencing on the PacBio system. Genomes were assembled by RS HGAP Assembly v3.0 and polished with Quiver. Complete genome sequences were annotated using the Rapid Annotation using Subsystem Technology (RAST).

Genomic relatedness was evaluated by different algorithms for genome-to-genome comparison. Average nucleotide identity (ANI) based on BLAST (ANIb) and MUMmer (ANIm) values were calculated in the JSpeciesWS online server (Richter et al., 2016). Digital DNA–DNA hybridization (dDDH) values were computed with Genome-to-Genome Distance Calculator (GGDC) version 2.1 (Meier-Kolthoff et al., 2013). OrthoANI values were determined using the Orthologous Average Nucleotide Identity Tool (OAT) (Lee et al., 2016). Genome-based phylogenetic tree was constructed using an up-to-date bacterial core gene set (UBCG) consisting of 92 genes (Na et al., 2018).

## Results and Discussion

### 16S rRNA Phylogeny

**FIGURE 1:**
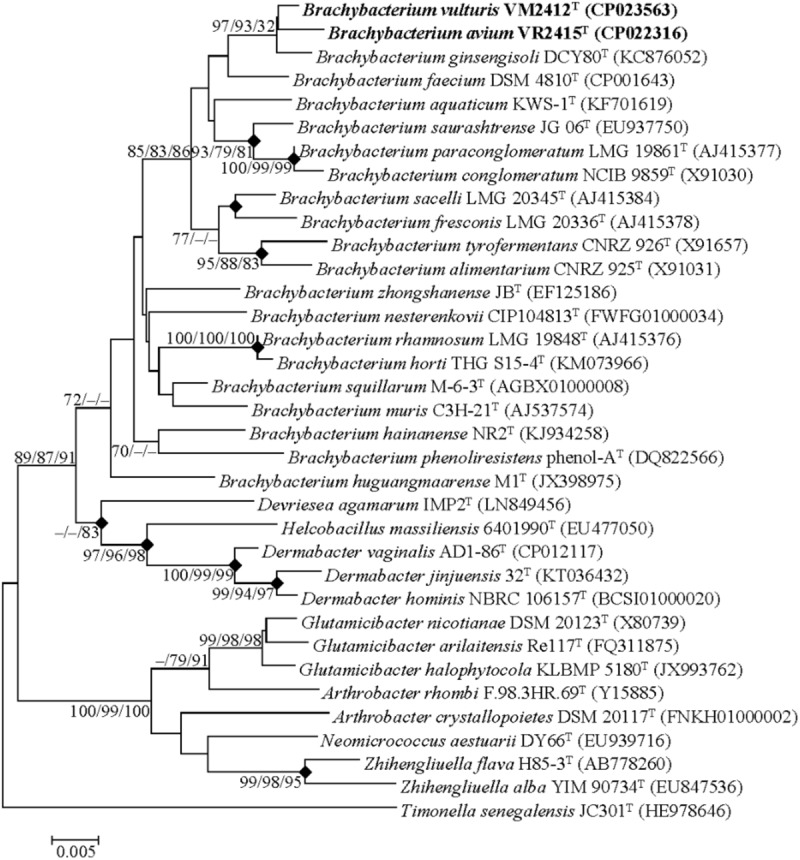
Neighbor-joining tree based on the 16S rRNA gene sequences. *Timonella senegalensis* JC301^T^ was used as an out-group. Nodes overlapping in neighbor-joining, maximum likelihood and maximum parsimony trees are indicated by filled diamonds. Bootstrap values (NJ/ML/MP) >70% based on 1000 replications are presented at nodes. Bar, 0.005 substitutions per nucleotide.

Genome-derived 16S rRNA gene sequences of strains VM2412^T^ and VR2415^T^ were found to share high sequence similarities with those of *B. ginsengisoli* DCY80^T^ (98.7 and 98.4%, respectively) and *B. faecium* DSM 4810^T^ (98.3% for both strains). The two strains formed a monophyletic clade with *B. ginsengisoli* DCY80^T^ in the phylogenetic tree based on 16S rRNA gene sequences (**Figure 1**). The clade containing type strains of *Brachybacterium* species was distinct from those composed of the members of other genera within the family *Dermabacteraceae*. Furthermore, the four genera in the family *Dermabacteraceae* formed a clade distinct from representative members of the family *Micrococcaceae*. These results suggest that the 16S rRNA tree accurately reflects the phylogenetic relationships.

### Phenotypic Characteristics

The two strains were found to grow at 10-30°C, with optimum growth at 30°C. They could tolerate salinity up to 15% (w/v) and grow at pH 6-9. Their cells were observed to be coccoid, Gram-stain positive, non-motile and aerobic. Colonies were observed to be 0.5-1 mm in diameter, ivory-colored, circular and low-convex with entire margins after incubation for 48 h on TSA.

Strains VM2412^T^ and VR2415^T^ were found to be catalase positive and oxidase negative. Further biochemical characteristics of strains VM2412^T^, VR2415^T^, *B. ginsengisoli* KCTC 29226^T^ and *B. faecium* JCM 11609^T^ were investigated using API kits.

Strain VM2412^T^ had the following enzyme activities: tryptophanase, urease, protease, esterase (C4), esterase lipase (C8), leucine arylamidase, valine arylamidase, crystine arylamidase, acid phosphatase, naphthol-AS-BI-phosphohydrolase, β-galactosidase, *β*-glucuronidase, α-glucosidase, β-glucosidase, *N*-acetyl-β-D-glucosaminidase and *α*-mannosidase. The strain could ferment glycerol, D-arabinose, D-ribose, D-xylose, D-galactose, D-glucose, D-fructose, D-mannose, dulcitol, D-mannitol, D-sorbitol, *N*-acetylglucosamine, amygdalin, arbutin, esculin, salicin, D-cellobiose, D-maltose, D-lactose, D-melibiose, D-saccharose, D-melezitose, D-raffinose, amidon, xylitol, gentiobiose, D-turanose, D-lyxose, D-tagatose, L-fucose, potassium gluconate and potassium 5-ketogluconate.

Strain VR2415^T^ had tryptophanase, protease, esterase (C4), esterase lipase (C8), leucine arylamidase, valine arylamidase, crystine arylamidase, acid phosphatase, naphthol-AS-BI-phosphohydrolase, β-galactosidase, α-glucosidase, β-glucosidase, *N*-acetyl-β-D-glucosaminidase and α-mannosidase enzyme activities. The strain could ferment glycerol, erythritol, D-arabinose, L-arabinose, D-ribose, D-xylose, D-adonitol, methyl β-D-xylopyranoside, D-galactose, D-glucose, D-fructose, D-mannose, D-mannitol, D-sorbitol, *N*-acetylglucosamine, amygdalin, arbutin, esculin, salicin, D-cellobiose, D-maltose, D-lactose, D-melibiose, D-saccharose, D-trehalose, D-melezitose, D-raffinose, amidon, glycogen, xylitol, gentiobiose, D-turanose, D-lyxose, D-tagatose, L-fucose, D-arabitol, L-arabitol, potassium gluconate, and potassium 5-ketogluconate.

Four strains used for the biochemical analysis were found to have tryptophanase, esterase (C4), esterase lipase (C8), leucine arylamidase, valine arylamidase, acid phosphatase, naphthol-AS-BI-phosphohydrolase, β-galactosidase, α-glucosidase, β-glucosidase, *N*-acetyl-β-D-glucosaminidase and α-mannosidase enzyme activities. All these strains could assimilate mannose, mannitol and potassium gluconate and ferment glycerol, D-arabinose, D-ribose, D-xylose, D-galactose, D-glucose, D-fructose, D-mannose, D-mannitol, D-sorbitol, *N*-acetylglucosamine, amygdalin, arbutin, esculin, salicin, D-cellobiose, D-maltose, D-lactose, D-melibiose, D-saccharose, D-melezitose, D-raffinose, amidon, xylitol, gentiobiose, D-turanose, D-lyxose, L-fucose, potassium gluconate and potassium 5-ketogluconate. Biochemical characteristics that differ between strains are shown in **Table 1**.

**Table 1:** Biochemical characteristics of *Brachybacterium vulturis* VM2412^T^ (1), *B. avium* VR2415^T^ (2), *B. ginsengisoli* KCTC 29226^T^ (3), and *B. faecium* JCM 11609^T^ (4).

Biochemical characteristics	1	2	3	4
**Enzyme activities (API ZYM/20 NE)**				
Urease	+	-	-	-
Protease	+	+	+	-
Crystine arylamidase	+	+	-	-
Trypsin	-	-	+	-
α-Galactosidase	-	-	+	+
β-Glucuronidase	+	-	-	+
**Assimilation of (API 20 NE)**				
Glucose	-	+	+	+
Arabinose	-	+	+	+
*N*-Acetylglucosamine	+	+	+	-
Maltose	-	+	+	+
**Acid production from (API 50 CH)**				
Erythritol	-	+	+	+
L-Arabinose	-	+	+	+
D-Adonitol	-	+	+	+
Methyl β-D-xylopyranoside	-	+	+	+
L-Sorbose	-	-	+	-
L-Rhamnose	-	-	+	+
Dulcitol	+	-	-	-
Inositol	-	-	+	-
Methyl α-D-mannopyranoside	-	-	-	+
Methyl α-D-glucopyranoside	-	-	+	+
D-Trehalose	-	+	+	+
Inulin	-	-	+	-
Glycogen	-	+	-	+
D-Tagatose	+	+	-	-
D-Fucose	-	-	+	-
D-Arabitol	-	+	+	+
L-Arabitol	-	+	+	+
Potassium 2-ketogluconate	-	-	+	-


*+, Positive; -, negative*

Strain VM2412^T^ was found to have anteiso-C_15:0_ (48.9%), anteiso-C_17:0_ (22.3%) and iso-C_16:0_ (13.5%) as major fatty acids (>10%). By contrast, strain VR2415^T^ was found to have anteiso-C_15:0_ (57.7%) and iso-C_16:0_ (13.7%) as predominant fatty acids (>10%). The fatty acid compositions of strains VM2412^T^, VR2415^T^ and closely related taxa are given in **Table 2**. The major fatty acid of all strains was identified as anteiso-C_15:0_, accounting for 42.9-57.7% of the total composition.

**Table 2:** Fatty acid profiles of *B. vulturis* VM2412^T^ (1), *B. avium* VR2415^T^ (2), *B. ginsengisoli* KCTC 29226^T^ (3), and *B. faecium* JCM 11609^T^ (4).

Fatty acid (%)	1	2	3	4
**Saturated acids**				
C_ 12:0_	-	1.9	0.5	1.3
C_ 14:0_	0.9	0.8	0.8	1.1
C_ 16:0_	2.9	1.0	3.3	3.3
**Unsaturated acids**				
C_19:0_ cyclo *ω*8*c*	-	2.9	-	-
**Branched acids**				
Iso-C_ 14:0_	0.8	2.4	1.0	0.5
Iso-C_ 15:0_	3.2	5.0	0.9	7.2
Anteiso-C_ 15:0_	48.9	57.7	51.3	42.9
Iso-C_ 16:0_	13.5	13.7	13.0	10.0
Iso-C_ 17:0_	1.4	0.5	0.5	4.2
Anteiso-C_ 17:0_	22.3	5.9	20.2	23.6
**Summed feature^∗^**				
4	0.6	0.4	1.5	0.3
8	3.6	5.2	4.6	2.7


The peptidoglycan of the two strains was found to contain alanine, glutamic acid, glycine and *meso*-diaminopimelic acid. Strain VR2415^T^ also contained aspartic acid within the peptidoglycan. Ribose, glucose, and galactose were found to be present as whole cell sugars. Polar lipids included phosphatidylglycerol (PG), diphosphatidylglycerol (DPG), phosphatidylethanolamine (PE, presumptively) and three unidentified glycolipids (**Supplementary Figures 1A,B**). In addition, strain VR2415^T^ produced an unidentified phospholipid. Both strains were found to have MK-7 as their predominant menaquinone.

Along with the closely related taxa *B. ginsengisoli* DCY80^T^ and *B. faecium* NCIB 9860^T^, phenotypic characteristics of the type strains of *B. squillarum* and *B. nesterenkovii* were investigated because they were used for genotypic analyses using their available genome sequences. All type strains of *Brachybacterium*, *Devriesea* and *Dermabacter* spp. are non-motile and contain *meso*-diaminopimelic acid within their cell wall. Differential characteristics between the type strains of the three genera are presented in **Table 3**. PE was tentatively detected only in strains VM2412^T^ and VR2415^T^.

**Table 3:** Differential characteristics of type strains of *Brachybacterium*, *Devriesea*, and *Dermabacter* species: 1, *B. vulturis* VM2412^T^; 2, *B. avium* VR2415^T^; 3, *B. ginsengisoli* DCY80^T^ (Hoang et al., 2014); 4, *B. faecium* NCIB 9860^T^ (Collins et al., 1988); 5, *B. squillarum* M-6-3^T^ (Park et al., 2011); 6, *B. nesterenkovii* 35^T^ (Gvozdyak et al., 1992); 7, *Devriesea agamarum* IMP2^T^ (Martel et al., 2008); 8, *Dermabacter vaginalis* AD1-86^T^ (Chang et al., 2016); 9, *D. hominis* S69^T^ (Jones and Collins, 1988).

Characteristics	1	2	3	4	5	6	7	8	9

**Isolation source**	**Fecal sample of a *Vultur gryphus***	**Fecal sample of a *Vultur gryphus***	**Soil of a ginseng field**	**Poultry deep litter**	**Salt-fermented seafood**	**Milk products**	**Liver of a female *Agama impalearis***	**Vaginal fluid of a Korean female**	**Forearm of healthy adults**
O_2_ requirement	Aerobic	Aerobic	Aerobic	Aerobic	Aerobic	Facultative anaerobic	Facultative anaerobic	Facultative anaerobic	Facultative anaerobic
Catalase	+	+	+	+	-	ND	+	+	+
Oxidase	-	-	-	-	-	ND	-	-	-
Cell wall sugars	Ribose, glucose and galactose	Ribose, glucose and galactose	ND	Glucose, galactose	ND	Glucose, galactose and rhamnose	ND	Ribose, glucose and galactose	ND
Polar lipids^†^	DPG, PG, PE, GL	DPG, PG, PE, GL, PL	DPG, PG, GL, PL	DPG, PG, GL, PL	DPG, PG, GL, PL	ND	PG, PL, GL	DPG, PG, GL, AL	DPG, PG, PL, DGDG
Major menaquinone	MK-7	MK-7	MK-7	MK-7	MK-7	MK-7	MK-8	MK-9	MK-9


*+, Positive; -, negative; ND, no data. ^†^DPG, diphosphatidylglycerol; PG, phosphatidylglycerol; PE, phosphatidylethanolamine; GL, unidentified glycolipid(s); PL, unidentified phospholipid(s); AL, unidentified aminolipid(s); DGDG, diglycosyldiacylglycerol*.

### Genome Properties and Genetic Relatedness

Whole genome sequences were obtained from strains VM2412^T^, VR2415^T^, and *B. ginsengisoli* DCY80^T^ in this study. The genome properties of nine strains used in this study are presented in **Table 4**. The genome of strain VM2412^T^ is comprised of one circular chromosome of 3,796,663 bp long with 70.8 mol% G+C content. The genome of strain VR2415^T^, with three assembled contigs, is 3,568,502 bp long with 70.4 mol% G+C content. The G+C contents (%) of the two strains are the lowest among all strains of current *Brachybacterium* species. Members of the genus *Brachybacterium* have higher G+C contents and larger genomes than the members of the genera *Devriesea* and *Dermabacter*. Strains VM2412^T^, VR2415^T^, *B. ginsengisoli* DCY80^T^, and *B. faecium* DSM 4810^T^ each have a total of 9 ribosomal RNA-encoding genes (three 5S rRNAs, three 16S rRNAs and three 23S rRNAs). The genomes of all type strains of the *Brachybacterium* spp. analyzed have 50 tRNA-encoding genes except strain VM2412^T^.

**Table 4:** Genomic traits of type strains of *Brachybacterium*, *Devriesea* and *Dermabacter* species used in this study.

Strain	bp	Contigs	G+C (mol%)	Protein	rRNA	tRNA	Accession #
*B. vulture* VM2412^T^	3,796,663	1	70.8	3402	9	51	CP023563
*B. avium* VR2415^T^	3,568,502	3	70.4	3344	9	50	CP022316
*B. ginsengisoli* DCY80^T^	3,953,253	1	71.6	3519	9	50	CP023564
*B. faecium* DSM 4810^T^	3,614,992	1	72.0	3139	9	50	CP001643
*B. squillarum* M-6-3^T^	3,191,479	8	72.8	2855	6	50	AGBX01
*B. nesterenkovii* CIP104813^T^	3,021,972	119	72.4	2672	3	50	FWFG01
*D. agamarum* IMP2^T^	2,958,880	1	60.1	2591	6	46	LN849456
*D. vaginalis* AD1-86^T^	2,392,314	1	62.6	2098	10	48	CP012117
*D. hominis* NBRC 106157^T^	2,155,270	28	63.2	1919	4	47	BCSI01


**FIGURE 2:**
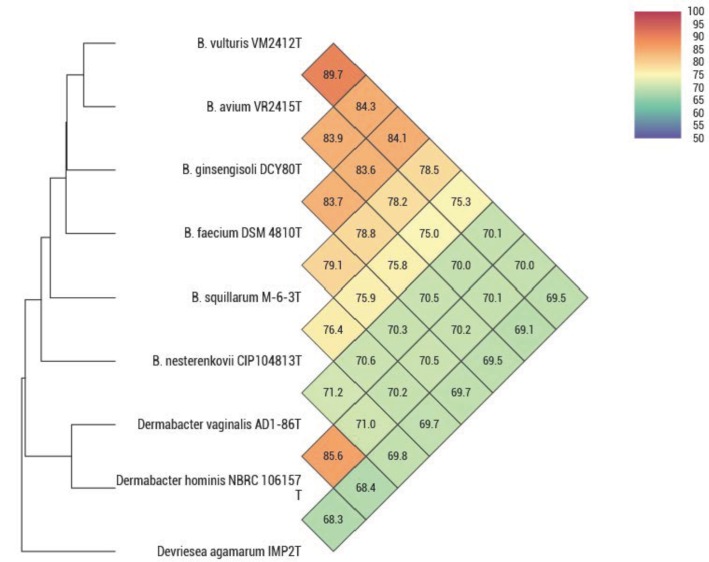
OrthoANI values between type strains of *Brachybacterium*, *Devriesea*, and *Dermabacter* species.

ANIb, ANIm, dDDH, and OrthoANI values were calculated to identify the genomic similarities of strains VM2412^T^ and VR2415^T^ to the seven strains of *Brachybacterium*, *Devriesea*, and *Dermabacter* species with available genome sequences. ANIb, ANIm, and dDDH values are presented in **Table 5** and OrthoANI values are shown in **Figure 2**. Strains VM2412^T^ and VR2415^T^ share 99.3% sequence similarity with each other based on 16S rRNA gene sequences whereas ANIb, ANIm, OrthoANI, and dDDH values between the two strains are 89.0-89.2, 90.3, 89.7, and 38.0%, respectively. The two strains show high 16S rRNA gene sequence similarities with *B. ginsengisoli* DCY80^T^ (98.7% for VM2412^T^ and 98.4% for VR2415^T^) and also have the closest genetic relatedness with *B. ginsengisoli* DCY80^T^ among the analyzed type strains of *Brachybacterium* species: 83.3-83.5% ANIb, 87.0% ANIm, 84.3% OrthoANI and 27.8% dDDH for VM2412^T^; 82.8–83.2% ANIb, 86.7% ANIm, 83.9% OrthoANI, and 27.2% dDDH for VR2415^T^. These findings support the classification of the strains as two distinct species according to cut-off values for species demarcation (95-96% for ANI and 70% for dDDH).

**Table 5:** ANIb, ANIm, and dDDH values between pairs of type strains of *Brachybacterium*, *Devriesea*, and *Dermabacter* species.

		1	2	3	4	5	6	7	8	9
**#ANIb**							
1	*B. vulturis* VM2412^T^	-	89.0	83.5	83.3	77.4	74.6	69.2	69.7	69.7
2	*B. avium* VR2415^T^	89.2	-	83.2	83.0	77.1	74.3	69.1	69.5	69.6
3	*B. ginsengisoli* DCY80^T^	83.3	82.8	-	82.7	77.4	74.8	69.1	69.9	70.0
4	*B. faecium* DSM 4810^T^	83.4	83.0	83.0	-	77.9	75.3	69.5	70.2	70.3
5	*B. squillarum* M-6-3^T^	78.1	77.8	78.5	78.5	-	76.1	69.7	70.3	70.4
6	*B. nesterenkovii* CIP104813^T^	75.0	74.7	75.3	75.6	76.2	-	69.7	70.9	70.7
7	*D. agamarum* IMP2^T^	69.0	68.9	69.2	69.3	69.5	69.7	-	68.3	68.1
8	*D. vaginalis* AD1-86^T^	69.7	69.5	70.0	69.8	69.9	70.9	68.5	-	85.2
9	*D. hominis* NBRC 106157^T^	69.8	69.7	70.0	70.1	69.9	70.9	68.3	85.2	-
**#ANIm**							
1	*B. vulturis* VM2412^T^	-	90.3	87.0	86.8	84.9	84.3	85.1	83.9	83.9
2	*B. avium* VR2415^T^	90.3	-	86.7	86.6	84.7	84.3	85.0	84.3	83.9
3	*B. ginsengisoli* DCY80^T^	87.0	86.7	-	86.7	85.0	84.3	85.4	84.2	83.7
4	*B. faecium* DSM 4810^T^	86.8	86.6	86.7	-	85.1	84.4	85.3	84.1	83.9
5	*B. squillarum* M-6-3^T^	84.8	84.7	85.0	85.1	-	84.8	85.2	84.7	84.1
6	*B. nesterenkovii* CIP104813^T^	84.3	84.3	84.3	84.4	84.8	-	84.9	84.0	83.8
7	*D. agamarum* IMP2^T^	85.1	85.2	85.3	85.3	85.2	84.9	-	85.2	85.3
8	*D. vaginalis* AD1-86^T^	83.9	84.3	84.0	84.1	84.7	84.1	85.1	-	88.2
9	*D. hominis* NBRC 106157^T^	83.9	83.9	83.7	83.9	84.0	83.8	85.2	88.2	-
**#dDDH**							
1	*B. vulturis* VM2412^T^	-								
2	*B. avium* VR2415^T^	38.0	-							
3	*B. ginsengisoli* DCY80^T^	27.8	27.2	-						
4	*B. faecium* DSM 4810^T^	27.5	27.0	26.6	-					
5	*B. squillarum* M-6-3^T^	21.4	21.2	21.7	21.9	-				
6	*B. nesterenkovii* CIP104813^T^	20.7	20.5	20.8	21.1	21.2	-			
7	*D. agamarum* IMP2^T^	22.4	22.8	24.4	23.0	22.9	23.2	-		
8	*D. vaginalis* AD1-86^T^	22.8	22.4	22.3	20.6	21.0	21.7	22.3	-	
9	*D. hominis* NBRC 106157^T^	21.0	20.3	22.0	21.1	20.1	20.9	23.1	29.6	-


**FIGURE 3:**
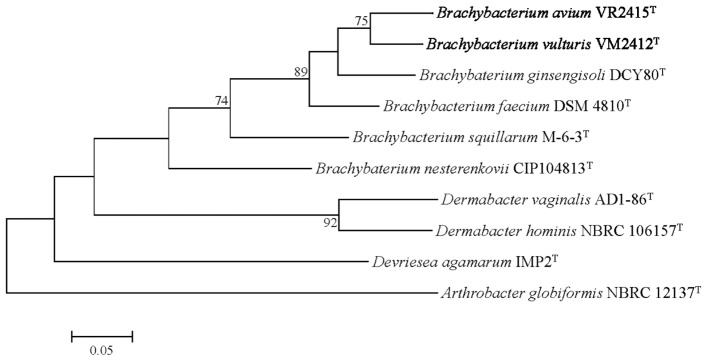
Genome-based phylogenetic tree constructed using an up-to-date bacterial core gene (UBCG) set. *Arthrobacter globiformis* NBRC 12137^T^ was used as an out-group. Numbers on nodes indicate the gene support index.

The UBCG phylogenetic tree verified the phylogenetic relationships derived from the 16S rRNA phylogeny and genetic relatedness, showing a monophyletic clade comprising strains VM2412^T^ and VR2415^T^ which is closely related to *B. ginsengisoli* DCY80^T^ (**Figure 3**).

### Comparative Genomic Analysis

Genome annotation was performed using the RAST annotation system. The following are comparisons of the genomic features derived from the genomes of strains VM2412^T^ and VR2415^T^. Common features shared by all nine strains used in the comparative genomic study are mentioned at the end of each section. Differences among characteristics derived from genome sequences of the members of the three genera are given in **Table 6**.

**Table 6:** Different genome-derived features of type strains of *Brachybacterium*, *Devriesea*, and *Dermabacter* species: 1, *B. vulturis* VM2412^T^; 2, *B. avium* VR2415^T^; 3, *B. ginsengisoli* DCY80^T^; 4, *B. faecium* DSM 4810^T^; 5, *B. squillarum* M-6-3^T^; 6, *B. nesterenkovii* CIP104813^T^; 7, *Devriesea agamarum* IMP2^T^; 8, *Dermabacter vaginalis* AD1-86^T^; 9, *D. hominis* NBRC 106157^T^.

	1	2	3	4	5	6	7	8	9
CRISPR/Cas system	+	-	-	-	+	+	-	-	+
Restriction-modification system	I, III	III	-	III	I, III	I	I	I	I, III
Polar lipids^†^	DPG, PG	DPG, PG	DPG, PG	DPG, PG	DPG, PG	DPG, PG	DPG, PG, PC	DPG, PG	DPG, PG
Sulfur metabolism						
Aryl sulfatase	+	-	-	-	-	-	-	-	-
Alkanesulfonate monooxygenase	-	-	+	+	-	-	-	-	-
Anaerobic respiratory reductases						
Ferredoxin reductase	+	+	+	+	+	-	+	-	-
Arsenate reductase	+	-	+	+	+	+	-	+	+
Dimethyl sulfoxide reductase	-	-	-	-	-	-	+	-	-
Nitrogen metabolism						
Nitrate reductase	+	+	-	+	+	+	+	-	-
Nitrite reductase	-	-	+	+	+	+	+	+	+
Urease	+	-	+	+	+	-	+	-	-
Stress response						
Betaine synthesis	+	+	+	+	-	+	-	-	-
Ectoine synthesis	+	+	+	+	-	-	-	-	-
Ferroxidase	+	+	+	+	+	-	-	-	-
Glutathione synthesis	+	-	+	+	+	-	-	-	-


*+, Positive; -, negative. ^†^DPG, diphosphatidylglycerol; PG, phosphatidylglycerol; PC, phosphatidylcholine.*

#### Defense Against Foreign DNA

Gene editing using CRISPR/Cas and restriction-modification systems is used for protection from foreign DNA. Strain VM2412^T^ has pre-crRNA processing-related genes *cse1-4, 5e* and *cas1, 3* (Gesner et al., 2011). Genes encoding the type III restriction-modification system are present in both strains, but the type I restriction-modification system is present only in strain VM2412^T^.

#### Motility

Genes belonging to the motility subsystem were not detected in the genomes of the two strains, consistent with the result of bacterial motility test using semi-solid agar. Similarly, the other strains analyzed do not encode genes that involved in the motility subsystem. Indeed, non-motility is one of the common features of the members of the family *Dermabacteraceae* (Stackebrandt, 2014).

#### Peptidoglycan Biosynthesis

Both strains contain gene *murE*, which catalyzes the addition of *meso*-diaminopimelic acid into peptidoglycan. This confirms the result of cell wall analysis using one-dimensional TLC. All the other strains analyzed also encode gene *murE* within their genomes. Members of the family *Dermabacteraceae* are known to have A4γ-type peptidoglycan, which contains *meso*-diaminopimelic acid (Stackebrandt, 2014).

#### Glycerophospholipid Metabolism

The enzyme for DPG synthesis is encoded in the genomes of the two strains. In both strains, enzymes for the biosynthesis of PG including phosphatidate cytidylyltransferase and CDP-diacylglycerol–glycerol-3-phosphate 3-phosphatidyltransferase are encoded but phosphatidylglycerophosphate is absent. Genes involved in the synthesis of phosphatidylcholine (PC), phosphatidylinositol (PI), phosphatidylserine (PS), and phosphatidylethanolamine (PE) are not present in the two strains while PE was detected by two-dimensional TLC. This latter identification needs to be confirmed; it is an atypical polar lipid in the majority of *Brachybacterium* species although PE has been detected in *B. aquaticum* (Kaur et al., 2016). All nine strains analyzed have genes for the synthesis of DPG and PG. The genome of *D. agamarum* IMP2^T^ includes the *cls* gene encoding cardiolipin (DPG) synthetase and the *pcs* gene encoding PC synthetase but DPG and PC were not detected in a previous characterization (Martel et al., 2008). This indicates potential discrepancies between genome-derived features and those obtained from some chemotaxonomic methods that will await detailed re-evaluation.

#### Sulfur Metabolism

In relation to metabolic pathways of sulfur compounds, strain VM2412^T^ possesses aryl sulfatase. However, none of the nine strains analyzed have genes for sulfur oxidation.

#### Anaerobic Respiration

Ferredoxin reductase, one of the anaerobic respiratory reductases, is present in the two strains. Furthermore, strain VM2412^T^ has arsenate reductase. In spite of the presence of anaerobic respiratory reductases, the strains are not able to grow under anaerobic conditions. Both strains possess respiratory nitrate reductase, which converts nitrate into nitrite. The ability to reduce nitrate was also detected using an API test kit. However, denitrification-related enzymes including nitrite reductase, nitric oxide reductase and nitrous oxide reductase are not present in any of the nine genomes analyzed. Nitrogenase involved in nitrogen fixation is also not encoded in the genomes of these nine strains.

#### Stress Response

Both strains contain enzymes involved in the biosynthesis of betaine from choline, including choline dehydrogenase (*betA*) and betaine aldehyde dehydrogenase (*betB*). Genes for ectoine biosynthesis (*ectB*, *ectA*, and *ectC*) are also present in the two strains. Osmolytes such as betaine and ectoine can protect cells against osmotic stress. Oxidative stress is suppressed by catalase, ferroxidase and manganese-dependent superoxide dismutase in the two strains. A synthetase for glutathione, known to be an important antioxidant, is encoded in the genome of strain VM2412^T^. Catalase and manganese-dependent superoxide dismutase-encoding genes are also present in the other strains analyzed.

#### Resistance to Antibiotics

Both strains encode β-lactamase that may provide resistance to *β*-lactam antibiotics. Strain VR2415^T^ has a gene encoding VanW, a vancomycin B-type resistance protein. All the other strains analyzed here also contain genes encoding β-lactamase.

## Conclusion

This study indicates that strains VM2412^T^ and VR2415^T^ represent two novel species of the genus *Brachybacterium* based on the results of phenotypic and genotypic analyses. Phylogenetic trees constructed using 16S rRNA gene sequences and core gene sets from whole genome sequences reveal the phylogenetic relationships between the isolates and other type strains of the genus *Brachybacterium*. In addition, this is the first study to reveal the taxogenomic relationships between members of the genus *Brachybacterium*. The following are descriptions of the newly isolated strains. The Digital Protologue database^1^ TaxoNumbers for strains VM2412^T^ and VR2415^T^ are TA00602 and TA00603.

### Description of *Brachybacterium vulturis* sp. nov.

*Brachybacterium vulturis* (vul.tu’ris. L. gen. n. *vulturis* from a vulture, the isolation source of the type strain).

Cells are coccoid, Gram-stain positive, non-motile, aerobic, catalase positive and oxidase negative. Colonies are 0.5-1 mm in diameter, ivory-colored, circular and low-convex with entire margins after 48 h incubation on TSA. Grows at 10-30°C, 0-15% (w/v) NaCl and pH 6-9. Optimal growth occurs at 30°C, 4% (w/v) NaCl and pH 7-8. Can reduce nitrates to nitrites and assimilate mannose, mannitol, *N*-acetylglucosamine and potassium gluconate. The major fatty acids are anteiso-C_15:0_, anteiso-C_17:0_ and iso-C_16:0_. The cell wall contains *meso*-diaminopimelic acid, alanine, glutamic acid and glycine and whole cell sugars are ribose, glucose and galactose. The polar lipids are composed of phosphatidylglycerol, diphosphatidylglycerol, presumptively phosphatidylethanolamine and three unidentified glycolipids. The predominant menaquinone is MK-7. The genomic G+C content of the type strain is 70.8 mol%.

The type strain is VM2412^T^ (=KCTC 39996^T^ = JCM 32142^T^), isolated from a fecal sample of an Andean condor (*Vultur gryphus*) in Seoul Grand Park, Gyeonggi-do, South Korea.

### Description of *Brachybacterium avium* sp. nov.

*Brachybacterium avium* (a’vi.um. L. gen. pl. n. *avium* of birds).

Cells are coccoid, Gram-stain positive, non-motile, aerobic, catalase positive and oxidase negative. Colonies are ivory-colored, circular, low-convex and 0.5-1 mm in diameter after 48 h incubation on TSA. Grows at 10–30°C (optimal growth at 30°C), 0-15% (w/v) NaCl [optimal growth at 3-4% (w/v) NaCl] and pH 6-9 (optimal growth at pH 6). Can reduce nitrates to nitrites and assimilate glucose, arabinose, mannose, mannitol, *N*-acetylglucosamine, maltose and potassium gluconate. The major fatty acids are anteiso-C_15:0_ and iso-C_16:0_. Peptidoglycan contains alanine, glutamic acid, glycine, aspartic acid and *meso*-diaminopimelic acid and ribose, glucose, and galactose are present as whole cell sugars. The major polar lipids are phosphatidylglycerol, diphosphatidylglycerol, presumptively phosphatidylethanolamine, three unidentified glycolipids and an unidentified phospholipid. The predominant menaquinone is MK-7. The genomic G+C content of the type strain is 70.4 mol%.

The type strain is VR2415^T^ (=KCTC 39997^T^ = JCM 32143^T^), isolated from a fecal sample of an Andean condor (*Vultur gryphus*) in Seoul Grand Park, Gyeonggi-do, South Korea.

## Author Contributions

All authors listed have made a substantial, direct and intellectual contribution to the work, and approved it for publication.

## Conflict of Interest Statement

The authors declare that the research was conducted in the absence of any commercial or financial relationships that could be construed as a potential conflict of interest.

## Footnotes

1 http://imedea.uib-csic.es/dprotologue/
