# Supplementary material for: Phenotypic and Genomic Properties of Brachybacterium vulturis sp. nov. and Brachybacterium avium sp. nov
Source: Front Microbiol. 2018 Aug 7;9:1809. doi: 10.3389/fmicb.2018.01809 (PMC6090031; doi:10.3389/fmicb.2018.01809)
Supplement: Supplementary file 1 [file Image_1.PDF]

Supplementary Material

Phenotypic and Genomic Properties of *Brachybacterium vulturis* sp. nov. and *Brachybacterium avium* sp. nov.

Euon Jung Tak, Pil Soo Kim, Dong-Wook Hyun, Hyun Sik Kim, June-Young Lee, Woorim Kang, Hojun Sung, Na-Ri Shin, Min-Soo Kim, Tae Woong Whon and Jin-Woo Bae*

Department of Life and Nanopharmaceutical Sciences and Department of Biology, Kyung Hee University, Seoul, Republic of Korea

***Correspondence:**
Jin-Woo Bae
baejw@khu.ac.kr

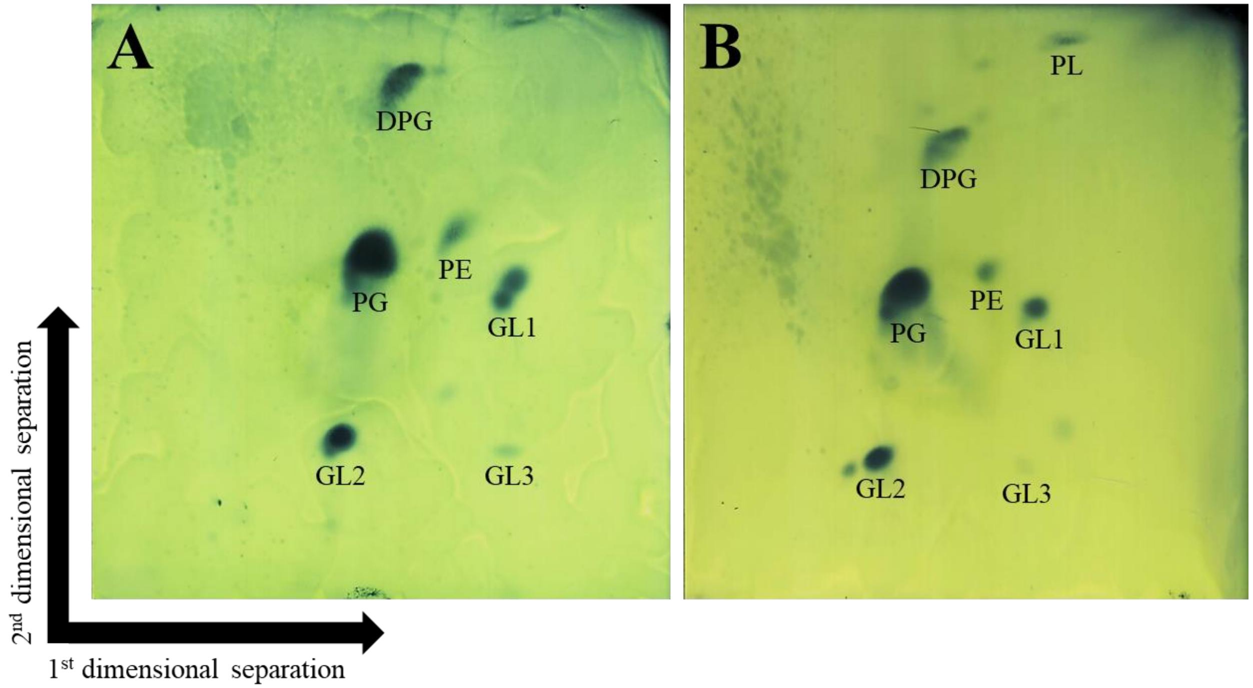

Supplementary Figure 1. Images of the polar lipid chromatograms of strains VM2412^{T} (**A**) and VR2415^{T} (**B**), visualized by spraying 10% (v/v) ethanolic molybdophosphoric acid and then heating at 180°C for 15 min. DPG, diphosphatidylglycerol; PG, phosphatidylglycerol; PE, phosphatidylethanolamine; GL, unidentified glycolipid; PL, unidentified phospholipid.
